# TP53 status and taxane-platinum versus platinum-based therapy in ovarian cancer patients: A non-randomized retrospective study

**DOI:** 10.1186/1471-2407-8-27

**Published:** 2008-01-29

**Authors:** Jolanta Kupryjanczyk, Ewa Kraszewska, Izabela Ziolkowska-Seta, Radoslaw Madry, Agnieszka Timorek, Janina Markowska, Jerzy Stelmachow, Mariusz Bidzinski, the Polish Ovarian Cancer Study Group (POCSG)

**Affiliations:** 1 Department of Molecular Pathology, the Maria Sklodowska-Curie Memorial Cancer Center and Institute of Oncology, Roentgena 5, 02-781 Warsaw, Poland; 2 Department of Gastroenterology and Hepatology, Medical Center for Postgraduate Education, Roentgena 5, 02-781 Warsaw, Poland; 3 Department of Gynecologic Oncology, the Maria Sklodowska-Curie Memorial Cancer Center and Institute of Oncology, Roentgena 5, 02-781 Warsaw, Poland; 4 Department of Pathology, IInd Faculty of Medicine, Warsaw Medical University and Brodnowski Hospital, Kondratowicza 8, 03-242 Warsaw, Poland; 5 Chair of Gynecologic Oncology, Medical University, Lakowa 1/2, 61-848 Poznan, Poland; 6 Chair and Department of Obstetrics, Gynecology and Oncology, IInd Faculty of Medicine, Warsaw Medical University and Brodnowski Hospital, Kondratowicza 8, 03-242 Warsaw, Poland

## Abstract

**Background:**

Taxane-platinum therapy (TP) has replaced platinum-based therapy (PC or PAC, DNA damaging chemotherapy) in the postoperative treatment of ovarian cancer patients; however, it is not always effective. TP53 protein plays a differential role in response to DNA-damaging agents and taxanes. We sought to define profiles of patients who benefit the most from TP and also of those who can be treated with PC.

**Methods:**

We compared the effectiveness of PC/PAC (n = 253) and TP (n = 199) with respect to tumor TP53 accumulation in ovarian cancer patients with FIGO stage IIB-IV disease; this was a non-randomized retrospective study. Immunohistochemical analysis was performed on 452 archival tumors; univariate and multivariate analysis by the Cox's and logistic regression models was performed in all patients and in subgroups with [TP53(+)] and without TP53 accumulation [TP53(-)].

**Results:**

The advantage of taxane-platinum therapy over platinum-based therapy was seen in the TP53(+), and not in the TP53(-) group. In the TP53(+) group taxane-platinum therapy enhanced the probability of complete remission (p = .018), platinum sensitivity (p = .014), platinum highly sensitive response (p = .038) and longer survival (OS, p = .008). Poor tumor differentiation diminished the advantage from taxane-platinum therapy in the TP53(+) group. In the TP53(-) group PC/PAC was at least equally efficient as taxane-platinum therapy and it enhanced the chance of platinum highly sensitive response (p = .010). However, in the TP53(-) group taxane-platinum therapy possibly diminished the risk of death in patients over 53 yrs (p = .077). Among factors that positively interacted with taxane-platinum therapy in some analyses were endometrioid and clear cell type, FIGO III stage, bulky residual tumor, more advanced age of patient and moderate tumor differentiation.

**Conclusion:**

Our results suggest that taxane-platinum therapy is particularly justified in patients with TP53(+) tumors or older than 53 years. In the group of patients ≤53 yrs and with TP53(-) tumors platinum-based therapy is possibly equally efficient. We provide hints for planning randomized trials to verify these observations.

## Background

First-line chemotherapy based on taxanes and platinum derivatives is a standard of care for ovarian cancer patients (taxane-platinum chemotherapy, TP) [1]; it replaced platinum-cyclophosphamide (PC) or other protocols of DNA damaging chemotherapy (PAC: platinum-doxorubicin-cyclophosphamide, or monotherapy), further called platinum-based therapy. However, taxane-platinum therapy is more costly, neurotoxic, and 20 to 30% of patients do not respond to this regimen [2,3]. These patients await alternative methods of treatment. The alternative might be a return to platinum-based therapy (PC or PAC) in some groups of patients. Defining clinicopathological and molecular profiles of tumors which show differential or equal response to these two basic modes of chemotherapy would allow for a customized treatment of ovarian cancer patients.

The agents used in platinum-based regimens exert a cytotoxic effect by damaging DNA [4,5]. This eventually leads to DNA strand-breaks and apoptosis. Apoptosis induced by DNA damage is dependent on tumor suppressor protein TP53 [6,7]. Taxanes exert a cytotoxic effect mainly by stabilising and inactivating microtubules responsible for the formation of mitotic spindle [8,9]. By continuous administration of taxol the cells are arrested in G2/M phases, mitosis cannot be completed and this activates apoptosis [8-11]. Apoptosis induced by taxanes is *TP53 *independent [12,13]. Thus, cisplatin and taxanes cause damage to other cell structures and activate different biochemical pathways.

Regarding the cisplatin treatment, impaired TP53 protein function (usually due to *TP53 *gene mutation) frequently contributes to resistance to this drug in cell line studies; there are also some experiments showing evidence for sensitisation or no effect of altered TP53 status on the cell outcome [14]. Among clinical studies on ovarian cancer patients, only a few addressed the issue of TP53 deficiency and tumor response to platinum-based chemotherapy [15-20]. Among these only Ferrandina et al. [17] showed a worse response in TP53-positive tumors in a multivariate analysis.

On the other hand, TP53 dysfunction may enhance tumor sensitivity to taxanes. Although not unanimously, it has been shown in experimental and pilot clinical studies that ovarian carcinomas with *TP53 *gene mutations show a better response to paclitaxel or paclitaxel containing regimens than tumors with normal TP53 [6,12,13,21-26]; however, these results have been obtained in univariate analyses only. To our knowledge, the role of TP53 dysfunction in taxane-platinum treated ovarian cancer patients has not yet been evaluated in a multivariate analysis; neither were the platinum-based and taxane-platinum regimens compared with each other with respect to the TP53 status. The findings presented above appear to provide a circumstantial evidence that taxane-platinum therapy is more efficient than platinum-based therapy in the group with TP53 dysfunction; however, little is known of the efficiency of the two types of regimens in the group without evidence of TP53 dysfunction.

Immunohistochemical detection of TP53 protein accumulation in tumor tissue is an inexpensive, fast and easy method of tumor screening for the TP53 gene missense mutation [27,28]; it is widely used in clinical studies for evaluation of TP53 status. Although tumors with nonsense and frameshift errors (which do not result in TP53 protein accumulation) cannot be detected by this method, the criterion of TP53 accumulation sequestrates tumors with the majority and with the most biologically potent gene alterations [29,30]. In our studies TP53 protein accumulation determined the clinical significance of other molecular factors [20,31,32].

We aimed to compare the efficiency of platinum-based and taxane-platinum chemotherapy in subgroups uniform as to TP53 status in a large group of ovarian cancer patients with advanced disease. TP53 status was determined by the lack or presence of TP53 accumulation in tumor tissue. According to our results TP53 protein accumulation is the main factor determining advantage of taxane-platinum over platinum-cyclophosphamide therapy. We are also reporting profiles of patients in whom platinum-cyclophosphamide therapy seems at least equally efficient.

## Methods

### Patients and tumors

The study was approved by the local ethics committee (decision ref. 49/2003). We studied archival material from 452 patients treated either with DNA damaging chemotherapy (PC: cisplatin-cyclophosphamide or carboplatin-cyclophosphamide, n = 193; PAC: PC with addition of doxorubicin, n = 60, as previously described) [20] or with standard taxane-platinum regimens (TP: paclitaxel or docetaxel [17 patients] with cisplatin or carboplatin, n = 199). In our previous analysis on PC and PAC treated patients we did not observe any difference in tumor response to these regimens [20], therefore both groups were naturally analyzed together.

The majority of patients from the PC/PAC group were operated on between the years 1990–1999, while from the TP group – between the years 2000–2005. Using the same criteria for the both chemotherapy groups optimal cytoreduction was more frequent in the TP group (41.8%) than in the PC/PAC group (25.3%), while suboptimal cytoreduction showed reverse proportions (33.2% versus 54.5%, respectively). The percentage of exploratory laparotomies was similar in the TP and PC/PAC group (23.1% and 20.2%, respectively); the number of patients who underwent intestinal surgery was higher in the TP group (9% versus 5.5% in the PC/PAC group).

The median age of patients was 53 years. In the younger patients group (≤53 yrs) optimal cytoreduction was performed more frequently than in the older patients group (>53 yrs) (36.2% versus 30.7%); a reverse proportion was observed for the exploratory laparotomy (17.6% in younger, 25.3% in older patients, chi-square p = 0.044). Suboptimal cytoreduction was performed in a similar percentage of younger and older patients (46.2% and 44.0%); intestinal surgery was more frequent in older patients (9.3% versus 5.3% in the younger group).

First-line chemotherapy consisted typically of 6–8 cycles in the PC/PAC group (mean 7) and 6 cycles in the TP group (mean 6). The number of cycles was lower if a patient experienced progression during the first line. In the PC/PAC treated group 90.5% of the patients completed 6 cycles of treatment; in the TP group the respective value was 95.9%.

Only patients treated with standard protocols of chemotherapy were accepted for the study. For the PC regimen it was 75 mg of cisplatin/m^2 ^or carboplatin (350 mg/m^2 ^or AUC6) and 750 mg of cyclophosphamide/m^2^, for PAC it was 50 mg of cisplatin/m^2^, 50 mg of doxorubicin/m^2 ^and 500 mg of cyclophosphamide/m^2^. Taxol, given in an 24-hour (135 mg/m2) or 3-hour infusion (175 mg/m2), or docetaxel (75 mg/m^2^) was followed by cisplatin (75 mg/m^2^) or carboplatin (AUC6).

For the PC/PAC group follow-up time ranged from 1.44 (two patients who experienced progression died before completion of the first-line chemotherapy) to 195 months (median 27); the respective values for the TP group were: 4.7–88.7 months (median 29.4). The short follow-up resulted from early patient's death. All surviving patients had at least a 6-month follow-up and all but six surviving patients had at least a 2-year follow-up after the completion of chemotherapy. Table 1 gives detailed material characteristics.

**Table 1:** Patient characteristics (detailed data on patients in subgroups related to TP53 status and the chemotherapy type, as well as the number of patients at risk of recurrence and death in each subgroup at different follow-up points).

	**All patients, N = 452**		**TP53(-) group, N = 186**		**TP53(+) group, N = 266**	
Chemotherapy	PC/PAC	TP	PC/PAC	TP	PC/PAC	TP
	N = 253	N = 199	N = 104	N = 82	N = 149	N = 117
Age						
Range	24–77	20–78	24–76	20–74	25–77	33–78
mean (st.dev.)	53.9 (10.6)	54.6 (11.2)	53 (11.3)	55 (11.8)	54.5 (10.1)	54.4 (10.8)
FIGO stage						
IIB, IIC	18 (7%)	10 (5%)	6 (6%)	6 (7%)	12 (8%)	4 (3%)
IIIA, IIIB	56 (22%)	28 (14%)	23 (22%)	10 (12%)	33 (22%)	18 (15%)
IIIC	145 (57%)	141 (71%)	60 (58%)	61 (74%)	86 (58%)	80 (68%)
IV	33 (13%)	20 (10%)	15 (14%)	5 (6%)	18 (12%)	15 (13%)
Residual Tumor Size						
0	53 (21%)	37 (19%)	18 (17%)	16 (20%)	35 (23%)	21 (18%)
>0 ≤ 2 cm	65 (26%)	81 (41%)	30 (29%)	35 (43%)	35 (23%)	46 (39%)
>2 cm	135 (53%)	81 (41%)	56 (54%)	31 (38%)	79 (53%)	50 (43%)
Histological Type						
Serous	199 (79%)	147 (74%)	72 (69%)	58 (71%)	127 (85%)	89 (76%)
Endometrioid, Clear cell	26 (10%)	13 (7%)	21 (20%)	8 (10%)	5 (3%)	5 (4%)
Undifferentiated	14 (5%)	21 (10%)	5 (5%)	7 (9%)	9 (6%)	14 (12%)
Other	14 (5%)	18 (9%)	6 (6%)	9 (11%)	8 (5%)	9 (8%)
Tumor grade						
G2	31 (12%)	26 (13%)	20 (19%)	15 (18%)	11 (7%)	11 (9%)
G3	158 (62%)	115 (58%)	59 (57%)	44 (54%)	99 (66%)	71 (61%)
G4	64 (25%)	58 (29%)	25 (24%)	23 (28%)	39 (26%)	35 (30%)
TP53 accumulation						
Negative	104 (41%)	82 (41%)	100%	100%	0	0
Positive	149 (59%)	117 (59%)	0	0	100%	100%
Response to chemotherapy						
complete remission	135 (53%)	131 (66%)	53 (51%)	51 (62%)	82 (55%)	80 (68%)
partial remission/no change^1^	52 (21%)	62 (32%)	18 (17%)	31 (39%)	34 (23%)	31 (27%)
progression	66 (26%)	6 (3%)	33 (32%)	0	33 (22%)	6 (5%)
Platinum sensitive	109 (43%)	112 (56%)	46 (44%)	42 (51%)	63 (42%)	70 (60%)
Platinum highly sensitive	43 (17%)	39 (20%)	18 (17%)	12 (15%)	25 (17%)	27 (23%)
Platinum resistant	144 (57%)	87 (44%)	58 (56%)	40 (49%)	86 (58%)	47 (40%)
Follow up time for alive patients	N = 30	N = 85	N = 11	N = 36	N = 19	N = 49
Range (months)	10–195	12.7–88.5	10–195	12.7–88.5	33–173.3	12.8–85.1
median	75.5	36.8	81.1	39.4	74.2	33.4
Number of patients at risk (OS)^2^						
1 year	212 (84%)	187 (94%)	82 (80%)	78 (95%)	130 (87%)	109 (93%)
2 years	137 (54%)	132 (74%)	54 (53%)	58 (75%)	83 (56%)	75 (74%)
3 years	90 (36%)	70 (51%)	38 (38%)	26 (47%)	52 (35%)	44 (53%)
4 years	61 (26%)	45 (39%)	23 (24%)	18 (39%)	38 (27%)	27 (39%)
5 years	44 (21%)	28 (31%)	17 (19%)	12 (34%)	27 (22%)	16 (29%)
Follow up for disease-free patients^3^	N = 23	N = 29	N = 10	N = 10	N = 13	N = 19
Range (months)	8.7–187.3	14.6–80.6	15.2–187.3	14.6–69.3	8.7–164.7	14.8–80.6
median	76.2	38.1	68.6	33.6	82.8	38.2
Number of patients at risk (DFS)^2^						
1 year	72 (54%)	75 (56%)	33 (62%)	27 (53%)	39 (49%)	48 (59%)
2 years	43 (33%)	39 (33%)	18 (35%)	12 (27%)	25 (31%)	27 (38%)
3 years	34 (26%)	23 (26%)	14 (27%)	7 (21%)	20 (25%)	16 (28%)
4 years	24 (20%)	14 (21%)	11 (23%)	5 (21%)	13 (18%)	9 (21%)
5 years	17 (17%)	6 (14%)	8 (21%)	2 (11%)	9 (15%)	4 (21%)

PC – cyclophosphamide and cisplatin, PAC – PC plus doxorubicin, TP-taxane-platinum therapy; ^1^we have combined these responses because it was not always possible to have objective measures of the disease in the restrospective study; OS-overall survival, DFS – disease free survival; ^2^based on Kaplan-Meier estimator, ^3 ^those who had complete remission only

### Selection of patients and reasons for exclusion

Medical records were reviewed centrally and all tumors were uniformly reviewed histopathologically according to the World Health Organization criteria [33] (Table 1). The material was carefully selected out of 899 cases submitted to meet several criteria, among them an adequate staging procedure that included bilateral salpingo-oophorectomy, total hysterectomy, omentectomy, aspiration of ascites or multiple cytologic washings, peritoneal biopsies including the diaphragm, complete abdominal exploration and pelvic and paraaortic node sampling (paraaortic lymphadenectomy has been performed in some our centers as a standard since the year 2000). If necessary the primary surgery was extended to bowel surgery and tumor debulking in the upper abdomen. However, not all centers performed such aggressive surgery. In patients who were not operated radically (suboptimal surgery or exploratory laparotomy) adequate staging procedure included at least the removal of adnexal masses, omentectomy or partial omentectomy or omentum biopsies, inspection and palpation of the peritoneal cavity, retroperitoneum and the whole abdomen, peritoneal biopsies, aspiration of ascites or multiple cytologic washings and subdiaphragmatic smears.

Among other criteria of inclusion were: no chemotherapy before staging laparotomy, International Federation of Gynecologists and Obstetricians stage IIB to IV disease [34], completed first-line chemotherapy (PC or PAC or TP, as above) and tumor tissue from the first laparotomy available.

Among the most common reasons of exclusion were: neoadjuvant chemotherapy, other chemotherapeutic regimens, early stage disease, inadequate staging procedure, lack of important data (residual tumor size, CA125 estimations), patient drop-out, inadequate histopathological diagnosis (a borderline tumor, a metastasis), interval debulking or secondary cytoreduction after completion of chemotherapy. Only a small percentage of patients underwent second-look procedure. Patients with a microscopic recurrence were excluded, because they received a second-line chemotherapy and could not be compared with patients who were observed only clinically and biochemically. Patients with well differentiated tumors were excluded, because apparently chemotherapy is of no benefit in low grade disease [35].

### Evaluation of clinical response to chemotherapy

Response to chemotherapy was evaluated retrospectively according to the World Health Organization response evaluation criteria [36]. The evaluation was based on data from medical records describing patient's clinical condition and CA125 levels in 3–4 week intervals. Complete remision (CR) was defined as disappearance of all clinical and biochemical symptoms of ovarian cancer evaluated after completion of first-line chemotherapy and confirmed at four weeks. Within the complete remission (CR) group we have identified platinum sensitive groups (PS, disease free survival longer than six months) and platinum highly sensitive groups (PHS, disease free survival longer than 24 months). Other tumors were described as platinum resistant [37] (Table 1).

### Immunohistochemical analysis

Immunohistochemical stainings were performed on paraffin-embedded material after heat-induced epitope retrieval (HIER). We used PAb1801 monoclonal antibody (1:3000, Sigma-Genosys, Cambridge, UK) for TP53 protein. This antibody detects both wild type and mutant TP53 proteins. In our experience it is highly effective in detecting tumors with TP53 gene missense mutations [27,28].

Deparaffinized sections were boiled in a citrate buffer (pH 6,0) at 700 Watts in a microwave 2 × 5 min. Non-specific tissue and endogenous peroxidase reactivity were blocked with 10% BSA and 3% H_2_O_2_, respectively. Tissue sections were incubated overnight at 4°C. Biotinylated goat anti-mouse IgG (1: 1500, cat. no.816), peroxidase conjugated streptavidin (1:500, cat. no. 309) (both from Immunotech, Marseille, France), and DAB were used as a detection system. Ovarian carcinomas with and without *TP53 *gene missense mutation were controls for TP53. Normal mouse IgG of the same subclass and concentration as the primary antibody also served as a negative control.

Semiquantitative evaluation of the immunohistochemical staining was performed by a pathologist (JK). TP53 protein accumulation was described as present (more than 10% of positive cells, TP53-positive) or absent (TP53-negative). According to our experience from different studies, and with the HIER method applied in the detection of TP53, 10% is the optimal cut-off value for separation of tumors with and without a TP53 gene missense mutation [27,28,38].

### Statistical analysis

Associations between protein expression and clinicopathological variables were studied by chi-square test. Overall survival and disease free survival analyses were performed with the Kaplan-Meier method and the multivariate Cox's proportional hazards models. Tumor response to chemotherapy was evaluated in the multivariate logistic regression model. Significant parameters were selected by stepwise elimination from the model. The analysis was performed in all carcinomas, and separately in tumors with [TP53(+)] and without TP53 accumulation [TP53(-)] [20]. In the smaller TP53(-) group (n = 186) a two-sided log rank test achieved 80% power at a 0,05 significance level to detect a difference of 15% of patients surviving 1 year between the two chemotherapy groups. All tests were two-sided and the level of significance was set at 5%.

#### Analysis of interaction between the evaluated chemotherapies and other factors

We also studied the relative efficiency of the main types of chemotherapy (TP versus PC/PAC) in various clinical and pathological subgroups. For example, not only patients with FIGO IIIC disease but also patients with FIGO IIIC disease treated with PC/PAC, and patients with FIGO IIIC disease treated with TP were compared to patients with FIGO IIB/C disease. These analyses were performed in the multivariate models. If the odds of achieving a given endpoint differed significantly between the both chemotherapy groups, it meant that one chemotherapeutic regimen was more efficient in the FIGO IIIC group (in other words it showed an interaction with FIGO IIIC).

#### Counting cumulated odds/hazards of achieving a given endpoint with the two chemotherapy regimens

PC/PAC and TP chemotherapy showed interaction with several clinicopathological parameters. In some patients the interaction was contradictory. For example, TP was more efficient in older patients, but less efficient in patients with undifferentiated tumors. To find out how various parameters and the two main chemotherapeutic regimens contribute to the final clinical effect we counted cumulated odds or hazards as in the example below.

Hazards of death in the TP53(+) group were as follows: the PC/PAC treated group served as a reference with HR 1.0 for all factors; risk of death for TP therapy was lower (HR 0.15) (see Table 2); for patients >53 years treated with TP the risk of death was lower (HR 0.58); for patients with grade 4 tumors treated with TP the risk of death was higher (HR 6.0). Thus, the cumulated hazard of death (with respect to these parameters only) in the TP53(+) group for a patient treated with PC/PAC (HR 1.0), older than 53 yrs (HR 1.0), with grade 4 tumors (HR 1.0) was 1.0 (1.0 × 1.0 × 1.0). The relative cumulated hazard of death in the TP53(+) group for a patient treated with TP (HR 0.15), older than 53 yrs (HR 0.58), with grade 4 tumors (HR 6.0) was 0.52 (0.15 × 0.58 × 6.0), i.e. it was nearly two times lower than with PC/PAC. All odds or hazards (OR, HR) were obtained in multivariate models.

**Table 2:** Taxane-platinum (TP) versus platinum-based therapy (PC/PAC) in the TP53-negative and TP53-positive group. Interactions between the therapies and clinicopathological factors (multivariate models: the logistic regression and Cox proportional hazards model)*

	**TP53(-) group, N = 186**		**TP53(+) group, N = 266**	
	OR or HR [95% C.I.]	P value	OR or HR [95% C.I.]	P value

**Complete remission (CR)**				
PC/PAC	-		1.0	
TP	-	>0.1	3.28 [1.22,8.83]	0.018
endometrioid, CCC treated with PC/PAC	1.0		-	
endometrioid, CCC treated with TP	>10 [0.92, +]	0.058	-	
**Platinum sensitivity (PS)**				
PC/PAC	-		1.0	
TP	-	>0.1	1.96 [1.14, 3.35]	0.014
**Platinum high sensitivity (PHS)**				
PC/PAC	1.0		1.0	
TP	0.006 [0.001, 0.29]	0.010	10.73 [1.14, +]	0.038
endometrioid, CCC treated with PC/PAC	1.0		-	
endometrioid, CCC treated with TP	18.37 [1.25, +]	0.033	-	
FIGO IIIA/B treated with PC/PAC	1.0		-	
FIGO IIIA/B treated with TP	161 [3.08, +]	0.012	-	
FIGO IIIC treated with PC/PAC	1.0		-	
FIGO IIIC treated with TP	133 [2.94, +]	0.012	-	
Residual tumor >2 cm treated with PC/PAC	-		1.0	
Residual tumor >2 cm treated with TP	-		5.95 [1.16, +]	0.032
Grade 3 tumors treated with PC/PAC	-		1.0	
Grade 3 tumors treated with TP	-		0.11 [0.01, 1.14]	0.065
Grade 4 tumors treated with PC/PAC	-		1.0	
Grade 4 tumors treated with TP	-		0.04 [0.003, 0.55]	0.016
**Disease free survival (DFS)**				
PC/PAC versus TP		>0.1		>0.1
**Overall survival (OS, risk of death)**				
PC/PAC	-		1.0	
TP	-	>0.1	0.15 [0.04, 0.60]	0.007
Age >53 treated with PC/PAC	1.0		1.0	
Age >53 treated with TP	0.51 [0.24, 1.08]	0.077	0.58 [0.32, 1.07]	0.080
Grade 3 tumors treated with PC/PAC	-		1.0	
Grade 3 tumors treated with TP	-		5.76 [1.44, 23.0]	0.013
Grade 4 tumors treated with PC/PAC	-		1.0	
Grade 4 tumors treated with TP	-		6.0 [1.43, 25.5]	0.015

CCC – clear cell carcinoma; *this table shows only parameters which interacted with the chemotherapies; the clinical endpoints were also associated with other clinicopathological factors or their categories (see Table 3)

## Results

### TP53 accumulation

Exactly the same percentage of ovarian carcinomas from the PC/PAC and taxane-platinum group were positive for TP53 protein accumulation (59%, Table 1). TP53 protein accumulation was associated with tumor grade 3 and 4 (p = 0.004) and with serous tumor type (p < 0.001); it did not associate with clinical factors or with clinical endpoints.

### Taxane-platinum versus platinum-based chemotherapy and clinical endpoints

#### Analysis of the TP53-negative group

In the TP53(-) group, complete remission (CR), platinum sensitivity, disease-free survival and overall survival were not influenced by the type of chemotherapy (Table 2). Platinum highly sensitive response (PHS) was more probable with PC/PAC regimen (OR 1.0 for PC/PAC, OR 0.006 for TP, Table 2). The latter was reflected mainly in patients with FIGO IIB/C and IV disease: patients with FIGO IIB/C had strikingly higher frequency and odds of PHS response when treated with PC/PAC (83% PHS versus 20% in the TP treated group), and the same was observed for FIGO IV disease (7% PHS versus 0 in the TP treated group) (cumulated OR 1.0 for PC/PAC, 0.01 to 0.006 for TP depending on histological tumor type, as below).

Analysis of the relative efficiency of the two types of chemotherapy revealed that patients with FIGO IIIA/B and IIIC disease, and with endometrioid and clear cell carcinomas would have higher chances of PHS response with taxane-platinum than with platinum-based therapy (Table 2); however, this effect was diminished by the generally lower odds of PHS with taxane-platinum therapy. Also, the odds of complete remission appeared higher after TP therapy in the group of endometrioid and clear cell carcinomas (p = 0.058, Table 2). However, some interactions were estimated on the basis of small patients groups and the odds had large confidence intervals; to determine their cumulative impact on reaching PHS or CR response in the TP53(-) group further studies would be necessary.

**Figure 1:**
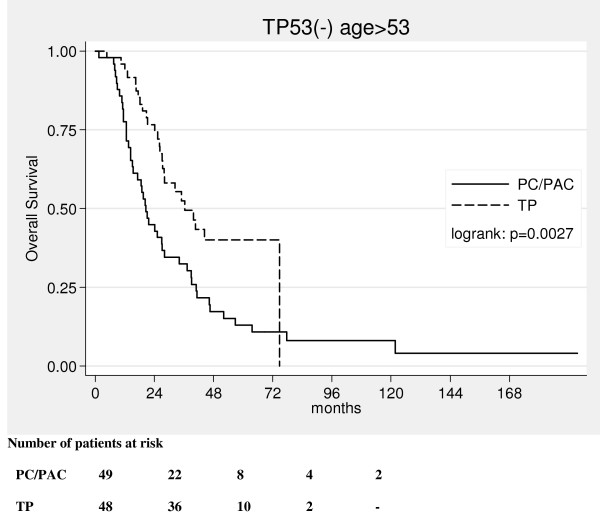
Kaplan-Meier curves for overall survival in the TP53(-) group in patients older than 53 years (median).

**Figure 2:**
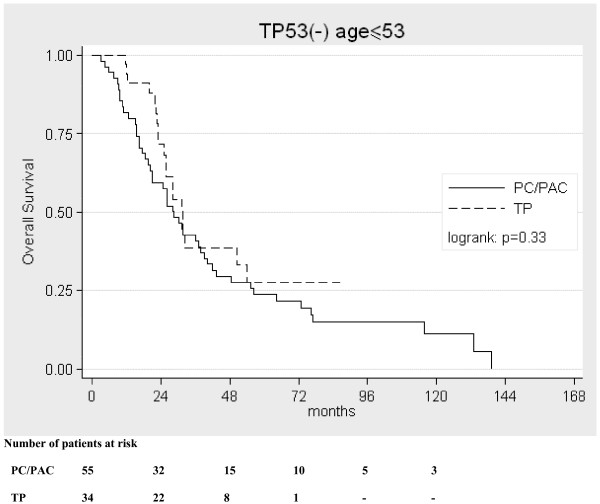
Kaplan-Meier curves for overall survival in the TP53(-) group in patients at the age of 53 years (median) or younger.

Overall survival curves have shown that in the TP53(-) group patient's age over median determined benefits from TP therapy (log rank: p = 0.0027, fig. 1). TP therapy in the group of patients ≤53 yrs did not seem to improve overall survival when compared with PC/PAC therapy (log rank: p = 0.37, fig. 2). In multivariate analysis there was a trend only (beyond the border of significance) suggesting that TP therapy might diminish the risk of death in patients older than 53 years (Table 2).

All clinicopathological factors influencing clinical endpoints in the TP53-negative and TP53-positive group are shown in Table 3.

**Table 3:** All clinicopathological factors influencing clinical endpoints in the TP53-negative and TP53-positive group.

	**TP53(-) group, N = 186**		**TP53(+) group, N = 266**	
	OR or HR [95% C.I.]	P value	OR or HR [95% C.I.]	P value

**Complete remission (CR)**				
PC/PAC	-		1.0	
TP	-	>0.1	3.28 [1.22,8.83]	0.018
Age ≤53 yrs	1.0		-	
Age >53	0.53 [0.27,1.02]	0.057	-	
Residual tumor 0	1.0		1.0	
Residual tumor ≤2 cm	0.15 [0.01,0.54]	0.004	-	
Residual tumor >2 cm	0.06 [0.02,0.24]	0.000	0.16 [0.094,0.29]	0.000
other histological types	1.0		-	
endometrioid, CCC	0.28 [0.08, 0.96]	0.044	-	
FIGO IIB-IIIC	-		1.0	
FIGO IV	-		0.35 [0.15,0.81]	0.014
**Platinum sensitivity (PS)**				
PC/PAC	-		1.0	
TP	-	>0.1	1.96 [1.14, 3.35]	0.014
Residual tumor 0	1.0		1.0	
Residual tumor ≤2 cm	0.15 [0.054, 0.45]	0.001	-	
Residual tumor >2 cm	0.08 [0.028, 0.23]	0.000	0.21 [0.126, 0.36]	0.000
FIGO IIB–IIIC	-		1.0	
FIGO IV	-		0.38 [0.158, 0.91]	0.031
**Platinum high sensitivity (PHS)**				
PC/PAC	1.0		1.0	
TP	0.006 [0.001, 0.29]	0.010	>10 [1.14, +]	0.038
FIGO IIB/C	1.0		1.0	
FIGO IIIA/B	0.06 [0.004, 0.68]	0.024	-	
FIGO IIIC	0.01 [0.001, 0.18]	0.001	0.47 [0.21,1.07]	0.075
FIGO IV	0.008 [0.000, 0.20]	0.003	0.13 [0.02,0.72]	0.021
Residual tumor 0	-		1.0	
Residual tumor ≤2 cm	-	0.012	0.41 [0.18,0.96]	0.04
Residual tumor >2 cm	-		0.09 [0.02, 0.37]	0.001
**Disease free survival (DFS)**				
PC/PAC versus TP		>0.1		>0.1
FIGO IIB-IIIB	1.0		1.0	
FIGO IIIC	2.17 [1.31,3.60]		-	
FIGO IV	3.04 [1.22,7.62]	0.003	2.20 [1.17,4.16]	0.014
Grade 3,4	-	0.017	1.0	
Grade 2	-		0.34 [0.16,0.72]	0.005
Residual tumor 0	-		1.0	
Residual tumor ≤2 cm	-		1.68 [1.07,2.64]	0.023
Residual tumor >2 cm	-		2.14 [1.34,3.39]	0.001
**Overall survival (OS)**				
PC/PAC	-		1.0	
TP	-	>0.1	0.15 [0.04, 0.60]	0.007
Age 53	1.0		1.0	
Age >53	1.5 [0.95, 2.25]	0.085	1.66 [1.14, 2.41]	0.008
Residual tumor 0	1.0		1.0	
Residual tumor ≤2 cm	2.48 [1.42,4.32]	0.001	1.72 [1.07,2.75]	0.023
Residual tumor >2 cm	3.13 [1.83,5.34]	0.000	2.43 [1.52,3.9]	0.000
other histological types	1.0		-	
endometrioid, CCC	1.76 [1.09,2.85]	0.02	-	
FIGO IIB/C	-		1.0	
FIGO IIIA/B	-		2.1 [0.92,4.76]	0.078
FIGO IIIC	-		2.58 [1.13,5.87]	0.024
FIGO IV	-		5.04 [2.06,12.27]	0.000

#### Analysis of the TP53-positive group

Taxane-platinum therapy in the TP53(+) group enhanced the chances of complete remission, platinum sensitivity and platinum highly sensitive response (PHS) when compared with PC/PAC therapy (Table 2). As to the odds of PHS, the greatest difference between the therapies was observed in patients with residual tumor above 2 cm (Tables 2 and 4). Also, patients with moderately differentiated tumors (G2) treated with taxane-platinum compounds had over a 10 times higher chance of PHS response than those treated with PC/PAC (final OR 10.7 for TP, 1 for PC/PAC, Table 4). On the other hand, tumor undifferentiation (grade 4) diminished the odds of the platinum highly sensitive response to TP therapy and enhanced the same to PC/PAC therapy (Table 2). This effect was particularly visible in patients with residual tumor ≤2 cm (cumulated OR 0.43 for TP, 1.0 for PC/PAC; Table 4). A similar impact of poor tumor differentiation (grade 3) was weaker (Tables 2 and 4).

**Table 4:** TP53-positive group. Cumulated odds of achieving platinum highly sensitive response with the two therapies in patients with various combinations of residual tumor size and tumor grade (the odds are based on multivariate models, table 2).

***Residual tumor (RT)***	***Tumor grade***	***Cumulated odds PC/PAC***	***Cumulated odds TP***
0	2	1	10.73
≤2 cm	2	1	10.73
>2 cm	2	1	63.84
			
0	3	1	1.18
≤2 cm	3	1	1.18
>2 cm	3	1	7.02
			
0	4	**1**	**0.43**
≤2 cm	4	**1**	**0.43**
>2 cm	4	1	2.55

The odds are higher for taxane-platinum therapy in all patients with residual tumor >2 cm or with grade 2 tumors. The odds are higher for platinum-based therapy in patients with grade 4 tumors and residual tumor 0–≤2 cm (bold). For other patients the odds for the two chemotheraputic regimens are similar.

**Figure 3:**
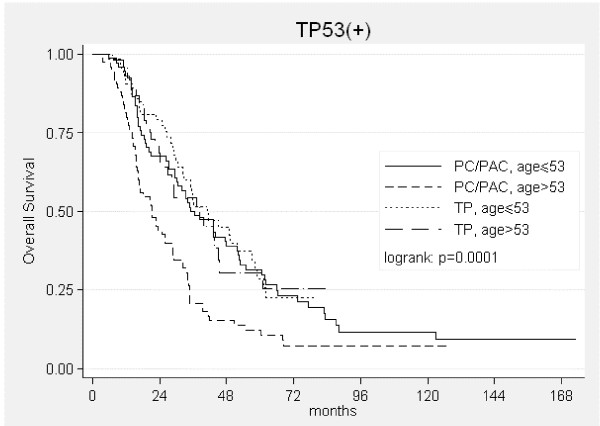
Kaplan-Meier curves for overall survival in the TP53(+) group in relation to patients' age.

**Figure 4:**
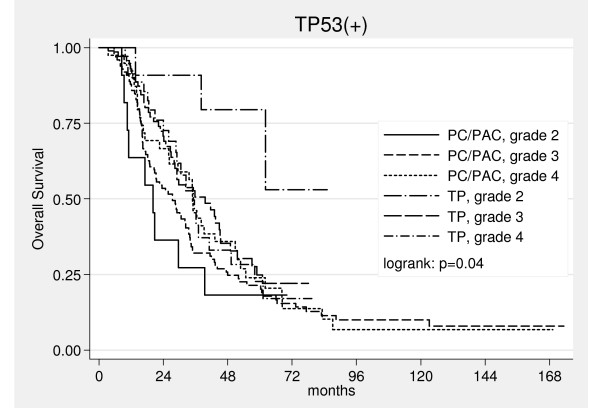
Kaplan-Meier curves for overall survival in the TP53(+) group in relation to tumor grade.

Risk of death was 6 times lower for patients treated with taxane-platinum than for those treated with PC/PAC (Table 2). There was a tendency for the highest advantage from taxane-platinum in patients over 53 years (p = 0.080, Table 2) (fig. 3) or with G2 tumors (fig. 4). Poor tumor differentiation (G3 or G4) relatively enhanced risk of death in patients treated with taxane-platinum compounds (Table 2).

Concomitant evaluation of three parameters influencing a given clinical endpoint (i.e. a product of odds ratios of age, grade, residual tumor size) might suggest that in the TP53(+) group there was a subgroup with higher chances of platinum highly sensitive response with PC/PAC therapy without compromising overall survival. This group was characterized by younger patient's age, residual tumor ≤2 cm, tumor grade 4 (Tables 4 and 5).

**Table 5:** TP53-positive group. Cumulated hazards of death in patients with various combinations of age and tumor grade (the hazards are based on multivariate models).

***Age***	***Tumor grade***	***Cumulated hazards PC/PAC***	***Cumulated hazards TP***
≤53	2	1	0.15
≤53	3	1	0.86
≤53	4	1	0.90
			
>53	2	1	0.08
>53	3	1	0.50
>53	4	1	0.52

The hazards of death are strikingly lower for taxane-platinum therapy in patients older than 53 years or with grade 2 tumors. For other patients the hazards for the two chemotheraputic regimens are close.

#### Analysis of the whole group

For comparison with data from the literature we also analyzed the whole patient group. Taxane-platinum therapy was more efficient than PC/PAC (OR 1.0) with regard to complete remission (OR for TP 1.57, 95% C.I. [1.03, 2.39], p = 0.035), platinum sensitivity (OR for TP 1.65, 95% C.I. [1.09, 2.50], p = 0.016) and overall survival (HR for TP 0.37, 95% C.I. [0.16, 0.81], p = 0.014); taxane-platinum therapy did not influence disease free survival and the probability of achieving platinum highly sensitive response (PHS). The latter was probably a result of contradictory associations between PHS and the therapies in the TP53(-) and TP53(+) groups.

Analysis of interactions revealed advantages or disadvantages of taxane-platinum therapy in some subgroups of patients, in accord with the results presented above, i.e. taxane-platinum therapy was more efficient than PC/PAC in patients with RT > 2 cm (platinum highly sensitive response, OR for TP 3.52, 95% C.I. [1.10, 11.27], p = 0.033), older than 53 years (overall survival, HR for TP 0.62, 95% C.I. [0.38, 0.97], p = 0.04), while it was less advantageous in cases with high tumor grade – G3 (overall survival, HR for TP 2.32, 95% C.I. [1.04, 5.17], p = 0.039) and G4 (overall survival, HR for TP 2.62, 95% C.I. [1.11, 6.18], p = 0.027).

## Discussion

There are four major randomized studies comparing platinum-based and taxane-platinum therapy (TP) in ovarian cancer patients and they present inconsistent results [1,39-41]. Nevertheless, taxane-platinum therapy has become a gold standard in the treatment of this disease. Its ineffectiveness in nearly a quarter of patients remains a real problem and identification of such patients prior to chemotherapy would allow for choosing alternative methods of treatment.

Our analysis of the whole group studied shows the superiority of TP over PC/PAC, and in this aspect it is in accord with the results of GOG 111 and OV-10 studies. However, we have possibly identified profiles of ovarian cancer patients who benefit most from first-line taxane-platinum therapy (TP), and also of those in whom platinum-based therapy (PC or PAC) appears at least equally efficient.

In our non-randomized retrospective study TP53 protein accumulation in tumor tissue was the main criterion of higher benefits from TP than from PC/PAC chemotherapy; in the TP53-negative group taxane-platinum therapy did not show any clear advantages over platinum-cyclophosphamide therapy. A similar observation was published by Zhao et al [42] who retrospectively analyzed 53 patients treated either with taxane-platinum or platinum-cyclophosphamide. Although their subgroups of patients were small they observed significantly higher rates of complete remissions with taxane-platinum therapy in the TP53(+) group only [42].

The observed advantage of taxane-platinum therapy in the group with TP53 accumulation is in accord with some earlier data from experimental and pilot clinical studies [21-23]. The clinical studies evaluating 38–48 patients treated with taxane-platinum compounds demonstrated better tumor response in the group with TP53 dysfunction [21,22]. Similar results were recently published by Ueno et al on larger group of 100 patients [24]. At a molecular level better sensitivity to paclitaxel in TP53 dysfunctional cells is not completely understood; a bypass of G1 checkpoint, an increase in G2/M arrest or loss of the TP53-dependent post-mitotic spindle checkpoint have been proposed as explanations [12,23].

In our study the patient's age over median (53 years in our material) appears as another important criterion of advantage of TP over PC/PAC therapy. This factor did not reach statistical significance in multivariate analysis, nevertheless, patients older than 53 years treated with PC/PAC had the worst survival rate. This result means a worse survival for older than for younger patients treated with PC/PAC, as it was shown in our previous multivariate analyses on the PC/PAC group only [20,31]; TP treated patients seem to have a similar survival rate in the both age groups [[43], Ziolkowska-Seta et al: TP53, BCL-2 and BAX expressions as predictors of ovarian cancer response to taxane-platinum therapy, submitted]. Possible mechanisms of the inferior results of cisplatin/cyclophosphamide treatment in the elderly may be due to slower metabolism of cyclophosphamide to active metabolites, higher cisplatin area under curve (AUC), higher toxicity and related morbidity; however, clinical analyses on age-related pharmacokinetics of these drugs are fragmentary and do not always confirm preclinical findings [44,45].

Of secondary importance in the criteria of benefits from taxane-platinum therapy observed in our study (in the TP53-positive group only) were bulky residual tumor and moderate tumor differentiation. This partially confirms the results of previous clinical studies showing that TP therapy improves survival in suboptimally debulked patients [1,40,46]. As TP therapy was generally much more efficient than PC/PAC in the TP53(+) group, its higher efficiency may be more visible in large tumor mass than in small or no tumor mass.

Spectacularly high efficacy of TP in moderately differentiated tumors and much lower in undifferentiated tumors (and concomitantly the opposite for platinum-based therapy) has not been reported previously. Poor tumor differentiation/undifferentiation appears as a factor significantly leveling the benefits from TP therapy in the TP53-positive group. While better effects of platinum-based therapy in undifferentiated tumors might be explained by loss of DNA repair in undifferentiated cells, we could not find a clear explanation for the observed associations between tumor grade and TP therapy. The mechanisms underlying taxol resistance include mutations in the β-tubulin gene [47-49], differential expression of β-tubulin isotypes [50,51], and altered microtubule dynamics [52]. The alterations may be more pronounced in less differentiated tumors. Several studies on cell lines demonstrated differences in the expression of beta-tubulin subtypes depending on cell differentiation; a higher stability of microtubules and lower activity of taxanes in differentiated cells was also described [53,54]. Shakuto et al observed better tumor suppression *in vivo *by paclitaxel in well differentiated than in moderately differentiated tumors [55].

We also observed that patients with endometriod and clear cell carcinomas treated with TP showed a better response than those treated with PC/PAC (in the TP53-negative group only). This may be due to the high expression of apoptosis inhibitor BCL-2 in these histological types and its inactivation by taxanes [20,56]. However, this association was not strong enough to show an effect on overall survival in patients younger than 53 years. An analysis of a larger group of endometriod and clear cell carcinomas should be performed with respect to the two types of therapy.

A striking observation was the much lower rate of responses to taxane-platinum therapy in FIGO II and IV disease in the TP53-negative group. These may be candidate groups for platinum-based therapy of choice. Due to the small number of cases further clinical studies focused on these subgroups including also FIGO IC-IIA disease may provide greater insight.

The results of our analysis are presented and discussed with the limitation that this is a non-randomized retrospective study. Therefore, there is some bias in our analysis related to differences in the material characteristics between the therapies. Nevertheless, the main focus of our study was the analysis across TP53 status and the results have been obtained in a multivariate analysis. Our study suggests the superiority of taxane-platinum therapy in the majority of patients with TP53(+) tumors and in any one older than 53 years. It also questions the necessity of taxane-platinum therapy in some subgroups of younger ovarian cancer patients. We provide hints for planning randomized trials to verify these observations.

## Conclusion

According to our non-randomized study, TP53 protein accumulation appears as the main factor determining benefits from taxane-platinum therapy in ovarian cancer patients. Another factor may be the more advanced age of the patient. Thus, our study confirms the advantage of taxane-platinum therapy in the majority of patients. Nevertheless, the prospect does exist for a return to platinum-cyclophosphamide therapy in some subgroups of patients, predominantly of those younger than 53 yrs with TP53-negative tumors. Our results give hints for randomized trials.

## Competing interests

The author(s) declare that they have no competing interests.

## Authors' contributions

JK conceived and coordinated the study, reviewed the pathology and immunohistochemistry, interpreted the results and drafted the manuscript. EK performed the statistical analysis with the analysis of interactions. IZ-S, RM, AT, JM, JS, MB participated in the design of the study, carried out patient recruitment, reviewed the clinical material centrally. Other members of the POCSG carried out patient recruitment from the platinum-cyclophosphamide-treated group. All authors read and approved the final manuscript.

## Pre-publication history

The pre-publication history for this paper can be accessed here:


